# Lamb Wave Probabilistic Damage Identification Based on the Exchanging-Element Time-Reversal Method

**DOI:** 10.3390/s24206516

**Published:** 2024-10-10

**Authors:** Zeyu Shu, Jian He, Muping Hu, Zonghui Wu, Xiaodan Sun

**Affiliations:** College of Aerospace and Civil Engineering, Harbin Engineering University, Harbin 150001, China; shuzeyu1994@hrbeu.edu.cn (Z.S.); hejian@hrbeu.edu.cn (J.H.); humuping@hrbeu.edu.cn (M.H.); wuzonghuib@hrbeu.edu.cn (Z.W.)

**Keywords:** exchanging-element time-reversal method, correlation analysis, Lamb waves, damage location, probabilistic damage imaging

## Abstract

The commonly used baseline-free Lamb wave damage identification methods often require a large amount of sensor data to eliminate the dependence on baseline signals. To improve the efficiency of damage localization, this paper proposes a new Lamb wave damage location method, namely the probabilistic exchanging-element time-reversal method (PEX-TRM), which is based on the exchanging-element time-reversal method (EX-TRM) and the probabilistic damage identification method. In this method, the influence of the damage wave packet migration on the correlation coefficient between the reconstructed signals of each sensing path and the initial excitation signal is analyzed, and the structure is divided into multiple regional units corresponding to the damage to locate damage. In addition, the influence of the number of sensing paths on the location accuracy is also analyzed. A method of damage probability imaging based on structural symmetry is proposed to enhance location accuracy in the case of sparse sensing paths. The experimental and simulation results verify that the method can achieve damage location with fewer excitation times. Moreover, this method can avoid the problem that the damage wave packet is difficult to extract, improve the efficiency of damage location, and promote the engineering application of the Lamb wave damage location method.

## 1. Introduction

Metal plate structures have been widely used in modern engineering because of their light weight, high strength, good stability, and convenient construction, such as aircraft manufacturing, ship engineering, bridge construction, pressure vessel design, etc. It is one of the basic components in the engineering field and also the basis for scholars to study the mechanical model [1,2,3]. During its long-term service process, the plate-like structure will gradually age due to complex environmental factors, such as high temperature, high pressure, and corrosion, as well as external loads, including wind, waves, impacts, and vibrations [4,5,6]. These will cause different degrees of damage, posing a significant threat to its safety and service life. As a kind of ultrasonic guided wave, Lamb wave has the advantages of low energy attenuation, high damage sensitivity, and long propagation distance. It is widely used in damage identification of metal plate structures [7,8,9,10].

When damage occurs in the structure, the propagation of Lamb waves in the structure will be affected, which is most directly manifested in changes to the waveform and amplitude of Lamb waves [11,12]. Therefore, the core of the active Lamb wave monitoring method is to extract the key features of damage from the Lamb wave signal captured by the sensor. These characteristics may include specific wave packets’ amplitude, phase, time, frequency, etc. [13,14,15,16]. The traditional Lamb wave damage identification method based on residual signal usually needs to obtain the signal of the structure in the healthy state in advance as the reference signal for subsequent analysis [17]. However, in practical engineering, many structures have been in service for years, making it impossible to obtain accurate reference information under healthy conditions. Moreover, various environmental factors (such as temperature changes, natural deformation of the structure, etc.) encountered during service continuously affect the signals, further reducing the stability of the already difficult-to-capture reference signals [18,19]. In such cases, if the traditional damage detection method based on residual signal comparison is still used, it may lead to a high false alarm rate, as any slight environmental change may be misjudged as structural damage.

Research has been conducted by some scholars on accurately obtaining reference signals, such as the research conducted by Sun et al. [20] on ambient temperature compensation, the self-referencing ultrasonic detection achieved by connecting fiber Bragg gratings (FBGs) at two different locations distant from the FBG proposed by Wee et al. [21], and the use of machine learning to establish a regression model related to undamaged structural conditions proposed by Malasco et al. [22]. However, it is still impossible to obtain the health reference signal completely and accurately, so researchers have begun to explore damage identification methods without reference signals. Among them, the time-reversal method (TRM) and probabilistic damage imaging method (PDI) have received extensive attention due to their unique advantages [23,24,25,26].

The time-reversal method does not require prior information about the sensors and the medium in advance. It can detect the location of damage by comparing the differences between the initial excitation signal and the reconstructed signal, or by directly analyzing the waveform of the reconstructed signal [27]. The probabilistic damage imaging method, on the other hand, does not require prior information or an understanding of complex propagation processes. It directly utilizes the characteristic information of the damage signals to calculate damage coefficients. Then, using specific algorithms, such as the reconstruction algorithm for probabilistic inspection of damage (RAPID) [28], to compute the region with the highest probability of damage occurrence, this determines the location of the damage [29].

The probabilistic damage imaging method is often used in conjunction with the time-reversal method, as they exhibit good compatibility together. The damage index of each sensing path can be obtained through the time-reversal method, and then the probabilistic damage imaging method can be utilized to visualize the distribution of damage probabilities across all grid points in the structure. Xia et al. [30] optimized the probabilistic damage index imaging algorithm based on delay-and-sum imaging by incorporating time-reversal Lamb wave technology with a novel probabilistic distribution function. This optimization not only improved the accuracy of damage location but also enhanced the quality of imaging. Miao et al. [31] proposed a dual-damage identification method for aluminum plates based on the integration of a sensor network, time-reversal Lamb wave signals, and a damage diagnosis imaging algorithm. This method does not rely on the reference signal and is suitable for locating multiple damages. Liu et al. [32] presented a Lamb wave damage identification method for detecting delamination damages in composite plates by combining the time-reversal method and reconstruction algorithm for probabilistic inspection of damage. This method integrates both summation fusion and multiplication fusion to provide a more comprehensive and accurate image of the damage. Additionally, it employs sensor array technology to address potential issues of uneven probability distribution caused by uneven density of sensor networks.

However, both the time-reversal method and the probabilistic damage imaging method often require measuring a large number of Lamb wave signals on the sensing path. The operation is cumbersome and inefficient, with higher demands on hardware in practical applications [33,34]. Current research primarily focuses on enhancing the accuracy of damage identification, while relatively less attention has been paid to improving the efficiency of the identification process. In the virtual time-reversal (VTR) method proposed by Cai et al. [35], the two-step measurement process of time reversal only needs to measure the response signal from the actuator to the receiver, without actually performing the time-reversal process, instead of performing virtual execution through theoretical analysis. This method reduces the actual operation steps of time reversal but needs an accurate transfer function to be obtained. Watkins et al. [36] proposed the modified time-reversal method (MTRM), which maintains the functions of the actuator and receiver as unchanged during the two-step measurement process of time reversal. It avoids the cumbersome operation of repeatedly switching actuators, thereby simplifying the execution process of the time-reversal method, but it does not reduce the number of excitations. Additionally, scholars such as Gao et al. [37] utilized artificial neural networks to enhance data processing efficiency. They employed envelope features to represent damage information, reducing data dimensionality and the number of sensors required. However, this approach only increases the efficiency of damage information extraction and thus still has certain limitations.

The Lamb wave probabilistic damage identification method proposed in this paper introduces probabilistic damage identification into the EX-TRM. The exchanging-element reception method can incorporate the sensor signals received after the exchanging-element reception, which are not utilized by the traditional time-reversal method, into the damage identification framework. With one time-reversal excitation, damage can be located, significantly enhancing the efficiency of damage identification [38]. It uses the migration distance of the damage wave packet after the exchanging-element reception to locate the damage. Accurate calculation of the time difference between the peak of the damage wave packet and the focused main wave packet is required, but in practical engineering, accurate extraction of the damage wave packet is greatly affected by environmental factors and wave packet aliasing phenomena.

Therefore, this paper proposes the PEX-TRM for damage location. This method retains the advantage of the TRM, which does not require a reference signal while increasing the number of available damage data by the exchanging-element reception method. At the same time, it reduces the requirements for signal processing technology, making the method more flexible and convenient for practical applications.

In this paper, firstly, the influence of the damage wave packet migration on the focused main wave packet waveform is analyzed, and the internal relationship between this influence and the damage location, as well as the sensing path, is studied. Then, the deviation values (DVs) of the reconstructed signals along various sensing paths under different damage locations are calculated, and a systematic statistical analysis of the sensing path information affected by the damage is conducted. By dividing the structure into multiple regional units corresponding to potential damage locations, the damage location is performed based on the number and location of sensing paths affected by the damage. Finally, the influence of the number of sensing paths on the accuracy of damage location is analyzed. In the case of sparse sensing paths, the probability damage imaging method is introduced to refine the damage location results, and the number of sensing paths required for probability damage imaging can be reduced.

## 2. Theory

### 2.1. PEX-TRM

The PEX-TRM is a damage identification method that combines the EX-TRM with the probabilistic damage identification method. It uses the influence of the damage wave packet migration on the focused main wave packet after the exchanging-element reception to locate the damage, thereby eliminating the need for extracting the damage wave packet itself.

Assuming that the initial excitation signal Ω(t) is excited by the sensor *i* and is received by the sensor *j* after propagating in the medium, when the excitation signal propagates in the interior of the structure, a part of the signal propagates directly to the sensor *j* without any scattering or reflection, which is called direct wave. Another part of the signal wave encounters the damage area during the propagation process, and after the damage scattering, the damage scattering wave is generated. The receiving time of the sensor *j* to the direct wave and the damage scattering wave are denoted by $t_{ij}^{0}$ and $t_{ij}^{D}$, respectively. Similarly, after the excitation signal is mitted by the sensor *i*, the times for the direct wave and the damage scattering wave to reach the sensor *k* are denoted by $t_{ik}^{0}$ and $t_{ik}^{D}$, respectively. The process of the time reversal and the exchanging-element reception is as follows:

(1) The initial excitation signal Ω(t) is emitted by sensor *i*, and the response signal received by sensor *j* can be expressed as:

(1) $$P_{ij}=a_{ij}\Omega \left(t-t_{ij}^{0}\right)+b_{ij}\Omega \left(t-t_{ij}^{D}\right)$$

where $a_{ij}\Omega \left(t-t_{ij}^{0}\right)$ is the direct wave, and $b_{ij}\Omega \left(t-t_{ij}^{D}\right)$ is the damage scattering wave. *a_ij_* and *b_ij_* are the scattering coefficients of the direct wave and the damage scattering wave from sensor *i* to sensor *j*, respectively.

(2) Time reversal is performed on the response signal *P_ij_*, and the inversion time is *T*. The obtained time-reversal excitation signal re-emitted by sensor *i* can be expressed as:

(2) $$R_{ij}=a_{ij}\Omega \left(T-t_{ij}^{0}-t\right)+b_{ij}\Omega \left(T-t_{ij}^{D}-t\right)$$

(3) After the time-reversal excitation signal is re-emitted by sensor *i*, the exchanging-element reception method is adopted. The reconstructed signal received by sensor *k* can be expressed as:

(3) $$\begin{array}{c} R_{ijk}=a_{ik}a_{ij}\Omega \left(T-t_{ij}^{0}-\left(t-t_{ik}^{0}\right)\right)+a_{ik}b_{ij}\Omega \left(T-t_{ij}^{D}-\left(t-t_{ik}^{0}\right)\right)+\\ b_{ik}a_{ij}\Omega \left(T-t_{ij}^{0}-\left(t-t_{ik}^{D}\right)\right)+b_{ik}b_{ij}\Omega \left(T-t_{ij}^{D}-\left(t-t_{ik}^{D}\right)\right) \end{array}$$

where *a_ik_* and *b_ik_* are the scattering coefficients of the direct wave and the damage scattering wave from sensor *i* to sensor *k*, respectively. $a_{ik}a_{ij}\Omega \left(T-t_{ij}^{0}-\left(t-t_{ik}^{0}\right)\right)$ is denoted as wave packet 1, which is the direct–direct wave. $a_{ik}b_{ij}\Omega \left(T-t_{ij}^{D}-\left(t-t_{ik}^{0}\right)\right)$ is denoted as wave packet 2, which is the damage–direct wave. $b_{ik}a_{ij}\Omega \left(T-t_{ij}^{0}-\left(t-t_{ik}^{D}\right)\right)$ is denoted as wave packet 3, which is the direct–damage wave. $b_{ik}b_{ij}\Omega \left(T-t_{ij}^{D}-\left(t-t_{ik}^{D}\right)\right)$ is denoted as wave packet 4, which is the damage–damage wave.

Among them, wave packet 1 is the direct–direct wave, which has the largest amplitude among all signals and is the main component of the focused main wave packet, making it the easiest to identify and extract. Therefore, the relative positions of each wave packet in the reconstructed signal can be analyzed by calculating the relative difference in arrival times between wave packet 1 and other wave packets:

(4) $$\left\{\begin{array}{l} \mathrm{wave}\;\mathrm{packet}\;2:\Delta t_{12}=t_{ik}^{0}-t_{ij}^{D}-(t_{ik}^{0}-t_{ij}^{0})\\ \mathrm{wave}\;\mathrm{packet}\;3:\Delta t_{13}=t_{ik}^{D}-t_{ij}^{0}-(t_{ik}^{0}-t_{ij}^{0})\\ \mathrm{wave}\;\mathrm{packet}\;4:\Delta t_{14}=t_{ik}^{D}-t_{ij}^{D}-(t_{ik}^{0}-t_{ij}^{0}) \end{array}\right.$$

Since the positions of sensors are artificially set, and the propagation speed of Lamb waves in the medium can also be calculated or measured, both $t_{ij}^{0}$ and $t_{ik}^{0}$ can be obtained. In format (4), $\Delta t_{12}$ contains $t_{ij}^{D}$, which is related to the damage location but independent of the position of the sensor *k*. Therefore, it can be considered that the position of wave packet 2 relative to wave packet 1 is fixed. $\Delta t_{13}$ contains $t_{ik}^{D}$, so it is related to the distance from the damage to the sensor *i* and *k*. After the exchanging-element reception, wave packet 3 will shift with the change in the receiving sensor *k*. $\Delta t_{14}$ contains $t_{ij}^{D}$ and $t_{ik}^{D}$, so it is related to the distance from the damage to the sensors *i*, *j*, and *k*. Although it will also change with the receiving sensor *k*, its calculation process is more complicated, and the amplitude of wave packet 4 is relatively small, which is generally difficult to be used for damage identification.

**Figure 1:**
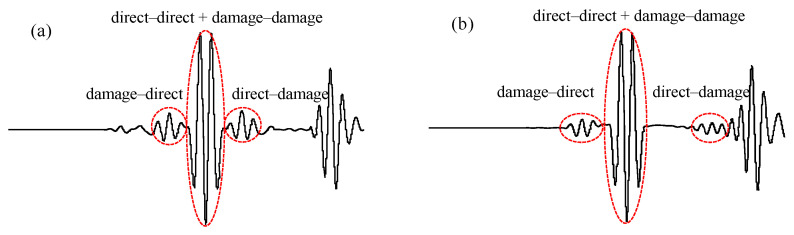
Schematic diagram of reconstructed signal: (**a**) without exchanging-element reception; (**b**) with exchanging-element reception.

The sensors can be arranged symmetrically according to the structure to make $t_{ij}^{0}=t_{ik}^{0}$ and $t_{ij}^{B}=t_{ik}^{B}$, so that the calculation process of the migration distance of the damage wave packet can be simplified, which is convenient for damage identification. The focused main wave packet is composed of the direct–direct wave and the damage–damage wave. Without exchanging-element reception, the damage–direct wave on the left side and the direct–damage wave on the right side are symmetrically distributed relative to the focused main wave packet. After the exchanging-element reception, the direct–damage wave on the right side will shift, which leads to the asymmetry of the waveform, as shown in Figure 1.

Therefore, the time difference $\Delta t_{13}$ between the direct–damage wave and the focused main wave packet in the reconstructed signal can be used to identify and locate the damage. $\Delta t_{13}$ is equal to the time difference between the propagation time of the damage scattering wave and the direct wave:

(5) $$\Delta t_{13}=\left(T-t_{ij}^{0}-\left(t-t_{ik}^{D}\right)\right)-\left(T-t_{ij}^{0}-\left(t-t_{ik}^{0}\right)\right)=t_{ik}^{D}-t_{ik}^{0}$$

**Figure 2:**
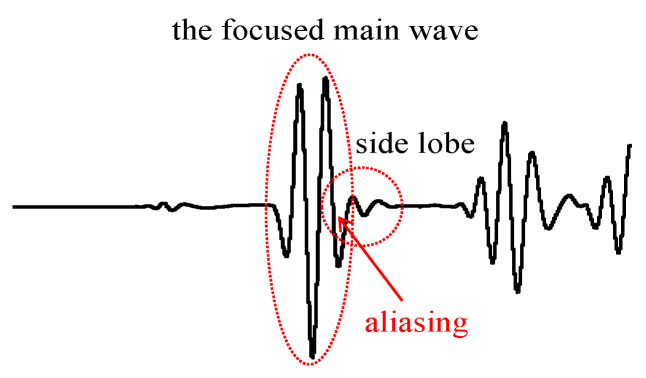
The aliasing phenomenon of the focused main wave packet and the side lobe signal.

Through one timereversal excitation process, multiple damage signals related to the exchanging-element reception sensors can be obtained, enabling the location of the damage position. Compared with the traditional time-reversal method, this approach reduces the number of time-reversal excitations and improves efficiency. However, the calculation of $\Delta t_{13}$ requires accurately extracting the peak time of the damage wave packet, which may not be possible due to experimental interference factors and wave packet aliasing. Therefore, this paper proposes a method for damage identification and location that utilizes the influence of the damage wave packet on the focused main wave packet. According to Equation (5), when the damage is close to a certain sensing path, $\Delta t_{13}$ is small, and the focused main wave packet is close to the direct–damage wave, leading to wave packet aliasing, as shown in Figure 2. Conversely, when the damage is far from a certain sensing path, $\Delta t_{13}$ is large, and the focused main wave packet is far away from the direct–damage wave, preventing wave packet aliasing. The focused main wave packet maintains its original characteristics without being affected by the damage wave packet.

Therefore, the positional relationship between the damage and each sensing path can be identified by analyzing the changes in the focused main wave packet of the reconstructed signals on each sensing path. If the focused main wave packet is not significantly affected, it can be judged that the damage is relatively far from that sensing path. On the other hand, if the focused main wave packet is significantly affected, the damage is relatively close to that sensing path. By comparing the changes in the focused main wave packets on different sensing paths, the sensing paths affected by the damage wave packet and the extent of the influence can be determined, thereby identifying the positional relationship between the damage and each sensing path.

Furthermore, it can be observed that the time difference between the arrival of the focused main wave packet (the direct–direct wave) and the damage sidelobe (the direct–damage wave) depends on the positions of the exchanging-element reception sensors, the excitation sensors, and the specific location of the damage and is independent of the position of the sensor that first receives the signal. Specifically, no matter which receiving sensor’s response signal is used to generate the time-reversal excitation signal, as long as the position of the damage in the plate remains unchanged, the arrival time and position of the damage wave packet in the reconstructed signals of each receiving sensor will remain consistent.

**Figure 3:**
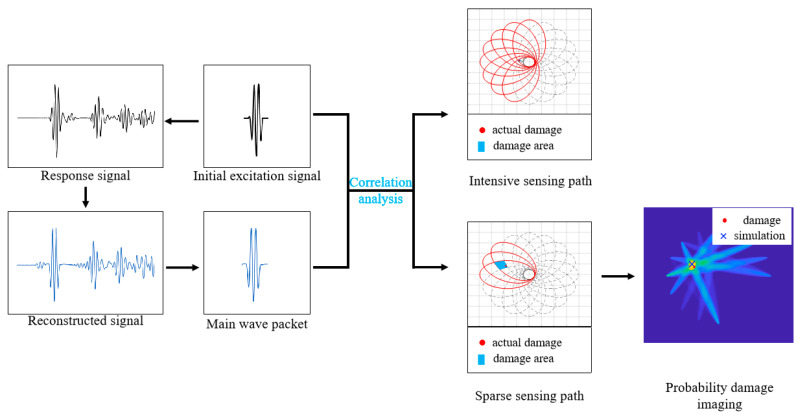
Damage location flow chart.

Therefore, a new Lamb wave damage location method, the PEX-TRM, is proposed. In this method, the damage location is identified by analyzing the correlation between the focused main wave packet in the reconstructed signal of each sensing path and the initial excitation signal. With one time-reversal excitation, if the sensing paths are dense, the damage can be directly located. If the sensing paths are sparse, the identified damage area is large, and the probabilistic damage imaging can be used to further locate the damage. At this time, this approach can also greatly reduce the invalid sensing paths and workload. The specific process of the damage location method is shown in Figure 3.

### 2.2. Correlation Analysis

The influence of the damage wave packet on the focused main wave packet is mainly reflected in the amplitude and waveform. The amplitude can be directly assessed by calculating the peak value change, but the peak signal is greatly affected by noise and medium properties. In contrast, changes in waveform are relatively stable and can be analyzed by calculating the correlation between the focused main wave packet and the initial excitation signal. The correlation between the focused main wave packet *V* (*t*) of the reconstructed signal and the initial excitation signal *V*′ (*t*) is calculated [39]:

(6) $$\rho_{v_{i},v_{i}\prime }(t)=\frac{n\sum v_{i}v_{i}\prime -\sum v_{i}\sum v_{i}\prime }{\sqrt{\left[n\sum {v_{i}}^{2}-{\left(\sum v_{i}\right)}^{2}\right]\left[n\sum v_{i}\prime^{2}-{\left(\sum v_{i}\prime \right)}^{2}\right]}}$$

where $\rho_{v_{i},v_{i}\prime }(t)$ denotes the similarity between *V* (*t*) and *V*′ (*t*). When the two signals are the same, $\rho_{v_{i},v_{i}\prime }(t)=1$. $\rho_{v_{i},v_{i}\prime }(t)$ is closer to 0, and the difference between the two signals is greater.

To better represent the difference between the reconstructed signal and the initial excitation signal, the concept of difference index (DII) is introduced:

(7) $${\mathrm{DII}}_{i}=1-\rho_{v_{i},v_{i}\prime }(t)$$

At the same time, the index of deviation value is introduced to quantify the deviation between the difference index of each sensing path and the average difference index of all sensing paths. The larger the deviation value, the greater the deviation between the difference index of the sensing path and the average value, which means that the path is more likely to be affected by the damage. The formula of the deviation value is as follows:

(8) $${\mathrm{DV}}_{i}=\frac{\left|{\mathrm{DII}}_{i}-\sum_{j=1}^{n}\frac{{\mathrm{DII}}_{j}}{n}\right|}{\sum_{j=1}^{n}\frac{{\mathrm{DII}}_{j}}{n}}$$

where *n* represents the number of sensing paths.

When using the exchanging-element reception method, the relative position relationship between the damage wave packet and the focused main wave packet will change, which is directly related to the distance between the damage and the sensing path. Specifically, when the damage is farther from the sensing path, the degree of separation between the damage wave packet and the focused main wave packet is greater, resulting in a gradual weakening of the influence of the damage wave packet on the focused main wave packet. When the peak of the damage wave packet no longer overlaps with the focused main wave packet, it can be considered that the influence of the damage on the focused main wave packet is already very weak. This means that the reconstructed signal of each sensing path is only sensitive to damage within a certain range nearby, and its damage identification ability has certain limitations, unable to sense damage in the structure infinitely. Based on this feature, the damage identification range of each sensing path can be defined as a specific area. This region can be roughly described as an elliptical region with the sensor pair of that path as its focus. According to which sensing paths’ reconstruction signals are affected, the structure can be divided into multiple regional units. These regional units correspond to different numbers and locations of the affected sensing paths to determine the location of the damage.

### 2.3. Probabilistic Damage Imaging

When conducting damage location through the method described in Section 2.2, the number of sensing paths serves as a crucial factor. The more sensing paths there are, the more precisely the regional units can be divided so that the location of the damage can be identified more accurately. However, due to the uneven density of the sensor network, when the number of sensing paths in the damage area is small, the damage can only be located to a certain range. To further enhance the accuracy of damage location, the probabilistic damage imaging method can be used to further locate structural damage on the basis of preliminary determination of damage scope.

The probabilistic damage imaging method is a damage identification method based on probability statistics. By analyzing the signals on multiple sensing paths, the damage probability of each sensing path can be calculated, and then the damage imaging is performed according to the sensor network formed by multiple sensing paths. And the damage index (DI) can be calculated by using the correlation between signals to characterize the degree of damage:

(9) $$\mathrm{DI}=1-\frac{n\sum_{i\in N}A(t_{i})B(t_{i})-\sum_{i\in N}A(t_{i})\sum_{i\in N}B(t_{i})}{\sqrt{\left[n\sum_{i\in N}A{\left(t_{i}\right)}^{2}-{\left(\sum_{i\in N}A(t_{i})\right)}^{2}\right]\left[n\sum_{i\in N}B{\left(t_{i}\right)}^{2}-{\left(\sum_{i\in N}B(t_{i})\right)}^{2}\right]}}$$

where $A(t_{i})$ and $B(t_{i})$ are the reference signal and the damage signal, *N* is the number of sampling points, and the range of DI value is [0, 1]. When the DI value is 0, there is no damage on the path. And the closer the sensing path is to the damage, the closer the DI value is to 1.

The monitoring area is gridded and divided into evenly distributed grid points. In this way, the continuous monitoring area can be discretized to facilitate numerical calculation and probability analysis. After gridding, each grid point represents a potential damage location. By calculating the damage probability of all grid points, visual damage imaging can be carried out, and the location with the highest damage probability is the damage location. When there are *n* sensing paths, the damage index on these sensing paths is considered to evaluate the possibility of each grid point as the damage location. The probability that the damage is located at the point (*x*, *y*) can be calculated as follows [40]:

(10) $$P(x,y)=\sum_{i=1}^{n-1}\sum_{j=i+1}^{n}{\mathrm{DI}}_{ij}s_{ij}(x,y)$$

(11) $$s_{ij}(x,y)=\left\{\begin{array}{c} \frac{\beta -R_{ij}(x,y)}{1-\beta },\beta >R_{ij}(x,y)\\ 0,\beta \leq R_{ij}(x,y) \end{array}\right.$$

(12) $$R_{ij}(x,y)=\frac{\sqrt{{\left(x-x_{i}\right)}^{2}+{\left(y-y_{i}\right)}^{2}}+\sqrt{{\left(x-x_{j}\right)}^{2}+{\left(y-y_{j}\right)}^{2}}}{\sqrt{{\left(x_{i}-x_{j}\right)}^{2}+{\left(y_{i}-y_{j}\right)}^{2}}}$$

where *p* (*x*, *y*) is the damage probability at the point (*x*, *y*). DI*_ij_* is the damage index of the sensing path between sensors *i* and *j*. (*x_i_*, *y_i_*) and (*x_j_*, *y_j_*) are the positions of the excitation sensor *i* and the receiving sensor *j*, respectively. *β* is an elliptical shape factor, which controls the size of the influence range of the damage index on the sensing path. If the value of *β* is too large, it will affect the resolution of the imaging. Conversely, if the value of *β* is too small, artifacts may occur, and the general value is 1.05.

## 3. Simulation Analyses

### 3.1. The Simulation Model

**Figure 4:**
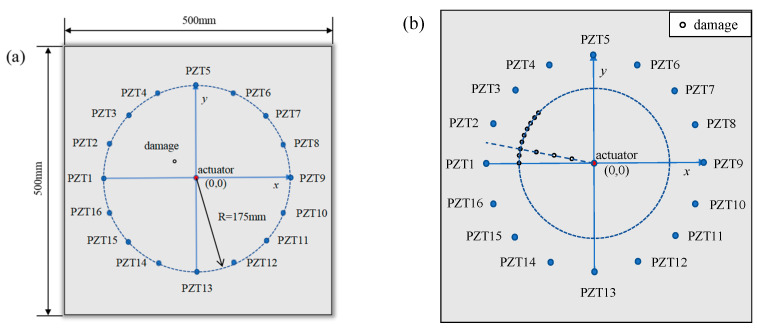
Geometric model: (**a**) sensor array; (**b**) damage location.

In this section, a 500 mm × 500 mm × 3 mm aluminum plate model is constructed using the finite element software Abaqus. A coordinate system is established with the center of the plate as the origin. The excitation sensor is located on the upper and lower surfaces at the origin, while a total of sixteen receiving sensors were arranged all on the same side of the plate. The excitation sensor (0, 0) in the center of the plate is taken as the center to form a receiving array, as shown in Figure 4a, and the coordinates of the receiving sensors are shown in Table 1. The initial excitation signal is a sinusoidal signal modulated by a Hanning window. The length of the sinusoidal signal is 3.5 signal periods, and the excitation frequency is 100 kHz. The sensing paths from the excitation sensor to PZT1–PZT16 are recorded as sensing paths 1–16.

The structural damage is set to a hole with a radius of 5 mm, where damage 1, 2, 3, and 4 are located at the middle of sensing paths 1 and 2, and the distances from the excitation sensor are 30 mm, 60 mm, 90 mm, and 120 mm, respectively. The damages 5 to 13 rotate clockwise around the center excitation sensor with 5.625° (skipping the position of damage 4), and the starting point is (−120.00, 0.00). The damages are located 120 mm away from the excitation sensor, as detailed in Figure 4b and Table 2.

### 3.2. Numerical Calculation

Firstly, the A0 mode Lamb wave is excited at the excitation sensor, and the response signal is obtained at the receiving sensors PZT1–PZT16. Then, the signal before the boundary reflection of the response signal (0.000127 s) is intercepted and inverted in the time domain to serve as the time-reversal excitation signal. Next, the time-reversal excitation signal is emitted at the excitation sensor, and the reconstructed signals are obtained at the receiving sensors PZT1–PZT16. Finally, the focused main wave packet of the reconstructed signal is extracted, and the correlation with the initial excitation signal is analyzed by Formulas (6)–(8).

**Figure 5:**
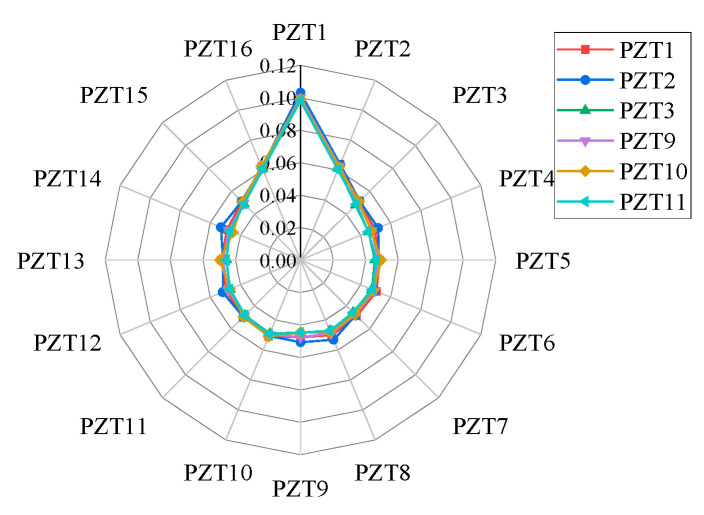
Difference indices of each sensing path.

The response signals from different receiving sensors are used to extract the time-reversal excitation signal. After the time-reversal excitation, the difference index of each sensing path is calculated. Taking damage 5 as an example, the response signals of PZT1, PZT2, PZT3, PZT9, PZT10, and PZT11 are selected for time-reversal excitation, and the difference indices of each sensing path are finally calculated, as shown in Figure 5.

It can be seen from Figure 5 that the difference index results of each sensing path are basically the same when different response signals are selected for time-reversal excitation, indicating that no matter which PZT response signal is used to extract the time-reversal excitation signal, the influence of damage on the sensing path can be effectively revealed. And the difference index of PZT 1 is about 0.1, which is significantly larger than that of PZT 2, PZT16 (about 0.06), and other PZTs (about 0.05), which means that sensing path 1 is the most sensitive to damage. Sensing paths 2 and 16 are also affected by damage but smaller than sensing path 1, so damage 5 is the closest to sensing path 1, followed by sensing paths 2 and 16.

**Figure 6:**
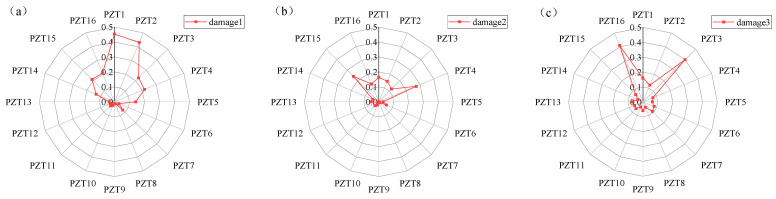

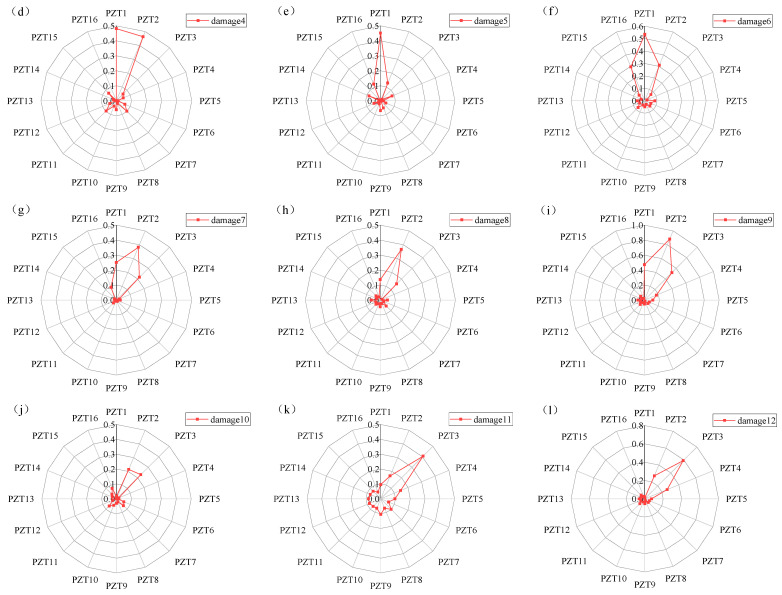
Deviation value of each sensing path: (**a**) damage 1; (**b**) damage 2; (**c**) damage 3; (**d**) damage 4; (**e**) damage 5; (**f**) damage 6; (**g**) damage 7; (**h**) damage 8; (**i**) damage 9; (**j**) damage 10; (**k**) damage 11; (**l**) damage 12.

When the response signal of PZT1 is intercepted for time reversal and used as the time-reversal excitation signal, the deviation values of each sensing path under 12 different damage locations are calculated, as shown in Figure 6.

The variation trend of the deviation value of each sensing path under different damage locations can be observed in Figure 6a–l. In the case of damage 1, the deviation values of sensing paths 1, 2, 3, 4, 5, 14, 15, and 16 are larger. In the case of damage 2, the deviation values of sensing paths 1, 2, 3, 4, 14, and 15 are larger. In the case of damage 3, the deviation values of sensing paths 1, 2, 3, and 14 are larger. In the case of damage 4, the relative deviation of sensing paths 1 and 2 is larger. It can be concluded that the farther the damage distance is from the excitation sensor, the less the number of sensing path’s focused main wave packet is affected by the damage.

In addition, in the case of damages 5, 8, or 12, the damage is located on a certain sensing path. The relative deviation of this sensing path is the largest, and the sensing paths on both sides also show a large deviation value, and the two values are not much different. In the case of damages 4 or 10, the damage is located in the middle of the two sensing paths. The deviation values of the two sensing paths are relatively large, with a small difference between them and values greater than those of other sensing paths. In the case of damages 6, 7, 9, or 11, the damage is located between the two sensing paths but closer to one of them. In this asymmetric case, the sensing path closest to the damage has the largest deviation value. In addition, the sensing paths on both sides of this sensing path also have large deviation values, and the deviation value of the sensing path closer to the damage is relatively larger. Therefore, it can be concluded that the closer the distance to the damage, the larger the relative deviation value of the sensing path.

### 3.3. Damage Location

**Figure 7:**
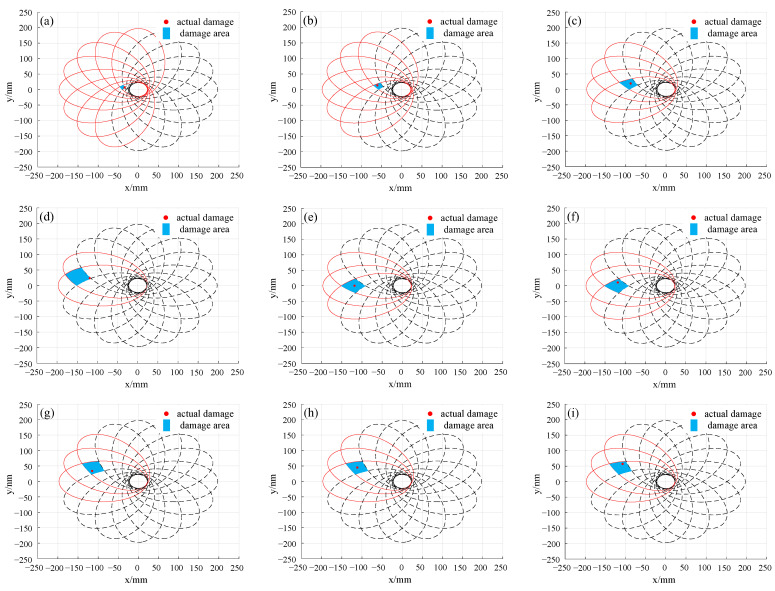

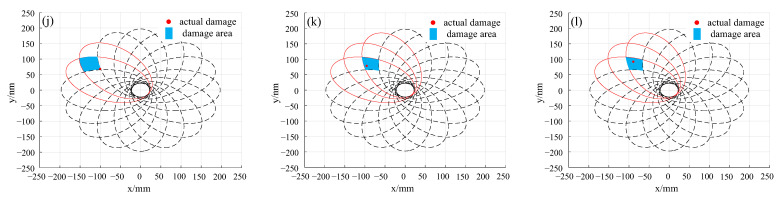
The results of damage location using deviation values: (**a**) damage 1; (**b**) damage 2; (**c**) damage 3; (**d**) damage 4; (**e**) damage 5; (**f**) damage 6; (**g**) damage 7; (**h**) damage 8; (**i**) damage 9; (**j**) damage 10; (**k**) damage 11; (**l**) damage 12.

To more accurately locate the damage, based on the results of numerical simulation and theoretical analysis, a threshold of deviation value is set to 0.1. When the deviation value is greater than the threshold, it is considered that the sensing path is affected by the damage. As shown in Figure 7, the sensitivity distribution of different sensing paths to damage and their corresponding damage unit can be clearly seen. The solid ellipses are the monitoring areas of sensing paths affected by the damage, while the dashed ellipses are the monitoring areas of sensing paths not affected by damage. When a significant change occurs in the difference index of the sensing paths within a certain area, it can be concluded that damage may exist in that area.

As can be seen from Figure 7, all damages 1 to 12 fall within the possible damage unit, which fully validates the effectiveness of locating damage positions by analyzing the number and location of sensing paths affected by the damage. Additionally, when the damage is closer to the excitation sensor, the more sensing paths are affected, the smaller the divided damage unit area, and the higher the recognition accuracy. Conversely, when the damage is farther away from the excitation sensor, fewer sensing paths are affected, leading to a larger divided damage unit, and further accurate damage location may be required. To improve the accuracy of damage location, the number of sensors can be increased to refine the damage unit.

### 3.4. Probabilistic Damage Imaging in Simulation

When the damage is located in an area with sparse sensing paths, probabilistic damage imaging methods can be used for further damage location. In traditional time-reversal probabilistic damage imaging, time-reversal excitation needs to be performed for each sensor pair to obtain reconstructed signals, and then the damage probability for each sensing path is calculated based on these reconstructed signals. This process can be cumbersome, especially when the number of sensors is large. However, if the approximate location of the damage has already been determined, the symmetry of the sensing path can be used to simplify the analysis process. Additionally, since the approximate damage location is known, the pseudo-phase interference unrelated to the damage can be eliminated, and the number of required sensing paths and the number of time-reversal excitations can be reduced.

**Figure 8:**
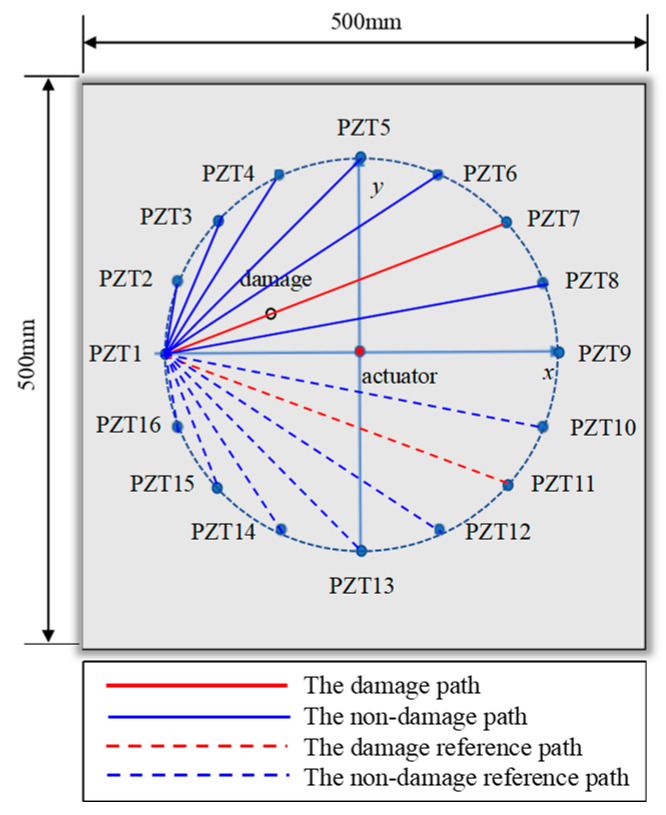
Symmetric sensing path diagram.

Based on the symmetry of the structure and sensor array, in a non-damaged state, sensors arranged symmetrically are exposed to similar mechanical environments and should capture identical structural vibration responses. Consequently, the signals received by the two symmetric paths are the same. If the signals on the two symmetric paths are highly consistent, this indicates that the structure is in a healthy state without any damage. However, when damage occurs, this symmetry is disrupted. The damage leads to a reduction in the local stiffness of the structure, further altering the characteristics of the vibration waves, such as amplitude, phase, or frequency, resulting in a decrease in the correlation between the signals on the two symmetric paths. As shown in Figure 8, among the pairs of sensing paths that are symmetrical with respect to the *x*-axis, the signal of sensing paths 1–7 undergoes significant changes due to the passage of damage, resulting in reduced signal correlation with its symmetrical sensing paths 1–11. In contrast, the correlation of signals from other symmetrical sensing paths remains largely unchanged.

**Figure 9:**
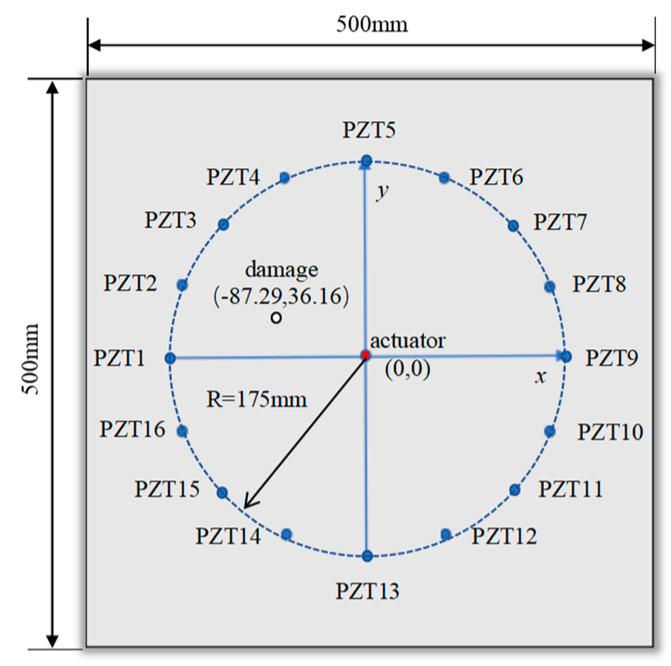
Structural model and sensor array.

Based on the model in Section 3.1, bilateral sensors are also arranged at PZT1, 5, 9, and 13, and the damage is set to a hole with a radius of 5 mm. The damage located at (−87.29, 36.16) is taken as an example for analysis, as shown in Figure 9.

**Figure 10:**
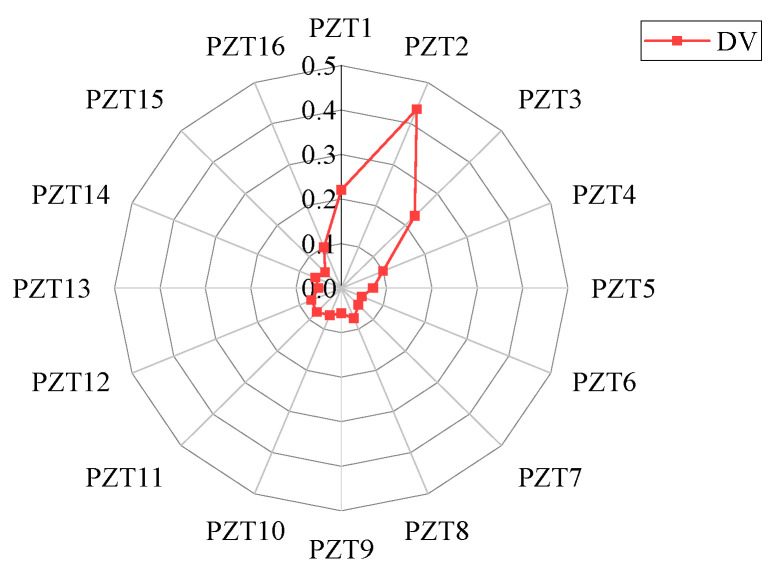
Deviation value of each sensing path in simulation.

**Figure 11:**
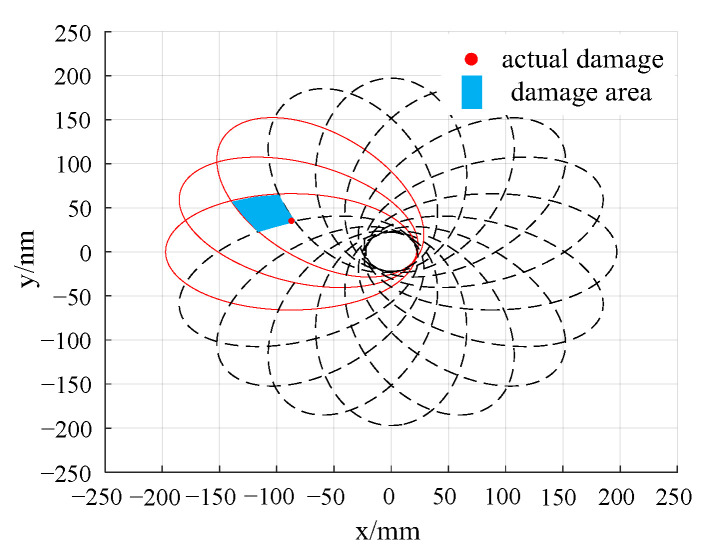
Damage location results using deviation values in simulation.

By performing the whole process of the EX-TRM and intercepting the response signal from PZT1 to perform time reversal as the time-reversal excitation signal, the reconstructed signal of PZT1−PZT16 can be obtained. Then, the deviation values of sensing paths 1 to 16 are calculated and presented in Figure 10. In the figure, the deviation value of sensing path 2 is the largest, which indicates that the focused main wave packet on sensing path 2 changes most significantly. The deviation values of sensing paths 1 and 3 are also greater than the threshold of 0.1, indicating that the two sensing paths also capture the signal changes related to the damage. The deviation values of other sensing paths are less than the threshold, and it can be judged that they are not affected by damage. So, only the deviation values of the sensing paths 1, 2, and 3 are greater than the threshold, while the other sensing paths are not affected by the damage, so the location of the damage is shown in Figure 11.

According to the symmetry of the structure and sensors, in the non-destructive state, the symmetrically arranged sensors are in a similar mechanical environment, and the same structural vibration response should be captured. Therefore, the signals received by the two symmetrical paths are the same. If the signals on the two symmetrical paths are highly consistent, this indicates that the structure is in a healthy state and no damage has occurred. When damage occurs, this symmetry will be broken. Damage will lead to a decrease in the local stiffness of the structure, which, in turn, changes the amplitude, phase, or frequency of the vibration wave, resulting in a decrease in the signal correlation on the two symmetrical paths.

**Figure 12:**
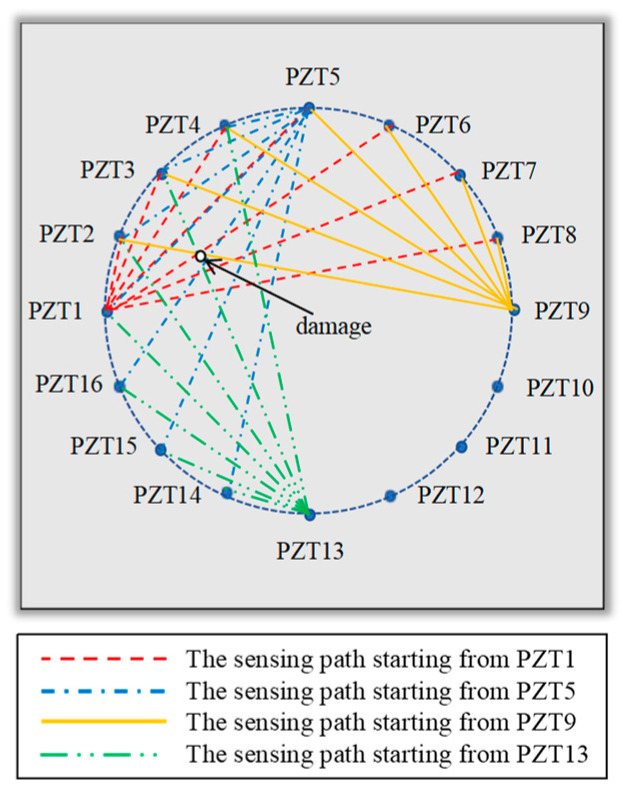
The sensing path near the damage.

The sensing path between the excitation sensor *i* and the receiving sensor *j* is recorded as the sensing path *i*-*j*. When the Lamb wave is emitted at PZT1, and the signal is received by other sensors because the known damage is located in the second quadrant, it is necessary to pay special attention to the sensing paths that pass through the second quadrant, so the damage indices of the sensing paths 1–2, 1–3, 1–4, 1–5, 1–6, 1–7, and 1–8 are obtained. Therefore, the correlation between symmetrical sensing paths 1–2 and 1–16, 1–3 and 1–15, 1–4 and 1–14, 1–5 and 1–13, 1–6 and 1–12, 1–7 and 1–11, and 1–8 and 1–10 signals is calculated by Formula (9). Similarly, excitations are also performed at PZT5, 9, and 13 to obtain the damage index of the corresponding sensing path, as shown in Figure 12. The traditional time-reversal probabilistic damage imaging method requires two excitations for each pair of sensors, totaling 240 excitations, and the calculation of damage probabilities for 120 sensing paths. However, this method only requires five excitations and the calculation of damage probabilities for only 28 sensing paths, significantly reducing the workload and improving efficiency.

**Figure 13:**
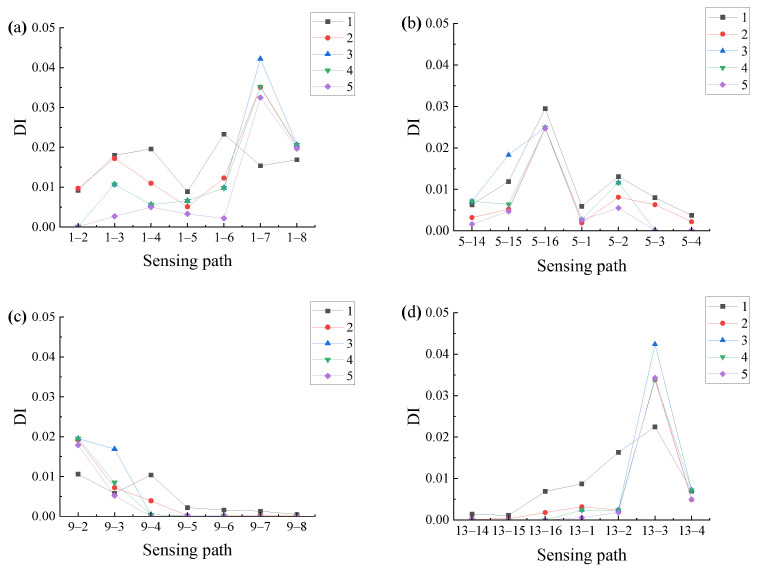
Damage indices of each sensing path emitted by different sensors in simulation: (**a**) PZT1; (**b**) PZT5; (**c**) PZT9; (**d**) PZT13.

When calculating the correlation of symmetrical sensing paths, the sensitivity of signals of different lengths to damage is different. Therefore, five different lengths of signals are taken for analysis: (1) fixed length 0.00025 s, (2) fixed length 0.00018 s, (3) the signal before the end of the first wave packet of each signal, (4) direct wave packet, and (5) the signal with an excitation wavelength centered at the peak point of the direct wave. By analyzing the signals of five different lengths, the damage index of each sensing path is obtained, as shown in Figure 13.

Through the distribution of the damage indices shown in Figure 13, it can be found that the sensing paths with larger damage indices are paths 1–7, 5–16, 9–2, and 13–3, respectively, which are all closer to the damage. When the length of the signal is (1) and (2), the result has a certain error because the longer signal length may contain more damage information, which cannot reflect the influence of a single damage wave packet on the signal, thus affecting the accuracy of the damage index. When the signal length is the case (3), (4), and (5), the damage index of the sensing path closer to the damage is relatively larger and easier to identify. But when the signal length is selected as the case (3), the damage index of the sensing path near the damage is not much different due to the long wave band before the direct wave, which will cause some interference. Comparing the signal of length (4) with length (5), the signal of length (5) is easier to extract, so the signal of length (5) is selected to analyze below.

**Figure 14:**
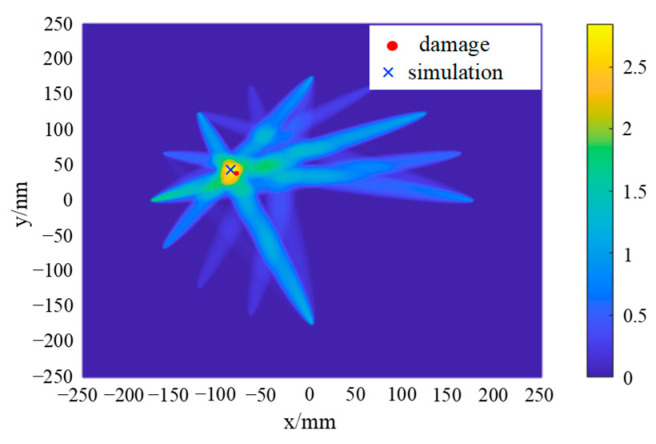
Probabilistic damage imaging results in simulation.

By calculating the deviation value of the sensing path 1 to path 16, it can be determined that the damage is located in the area shown in Figure 11. Therefore, it is only necessary to pay special attention to those sensing paths that pass through this damage area. The damage index of each sensing path passing through the second quadrant when emitted at PZT1, PZT5, PZT9, and PZT13 is analyzed, and the damage probability imaging is carried out, as shown in Figure 14.

In the damage imaging results presented in Figure 14, the highlighted area represents the region with the highest structural damage probability, with its central coordinates being (−88.50, 38.50). Compared with the actual damage location (−7.29, 36.16), the error is 1.39% in the *x* direction and 6.47% in the *y* direction, which proves the accuracy and reliability of the probabilistic damage imaging method. Compared with the traditional time-reversal probabilistic damage imaging method, the PEX-TRM significantly reduces the number of time-reversal excitations required. It effectively eliminates a large amount of invalid sensing path data, allowing for effective damage localization even with a substantial reduction in data processing.

## 4. Experiment

### 4.1. Damage Location Based on the Focused Main Wave Packet

**Figure 15:**
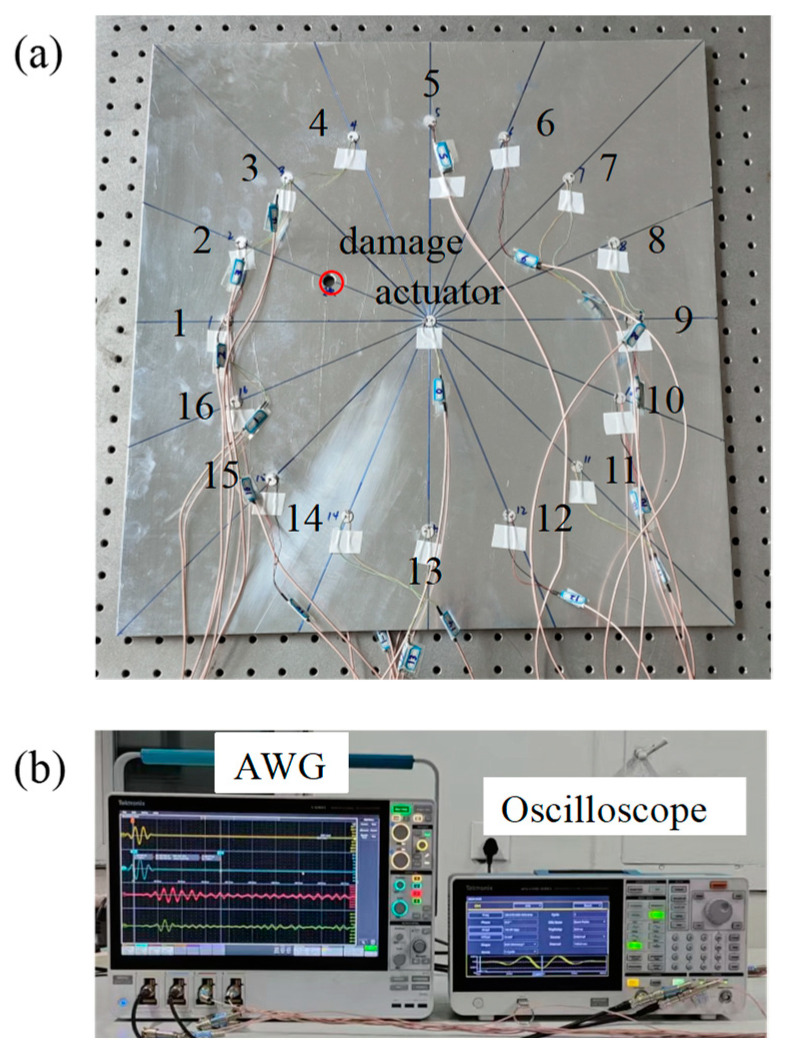
Experimental model: (**a**) sensors array; (**b**) arbitrary waveform generator and oscilloscope.

To verify the accuracy of the proposed damage location method, the same size aluminum plate as the numerical model (500 mm × 500 mm × 3 mm) is selected as the experimental subject. And piezoelectric sensors with a diameter of 10 mm and a thickness of 0.5 mm are selected as sensors. The sensors and damage are arranged according to the description in Section 3.4, as shown in Figure 15a. In the experiment, Tektronix arbitrary waveform generator (AWG) (Tektronix AFG31052, Tektronix Inc., Portland, OR, USA) and oscilloscope (MSO545 BW-350, Tektronix Inc., Portland, OR, USA) are used to emit and receive Lamb wave signals, as shown in Figure 15b. The initial excitation signal of the experiment is the same as that in the simulation.

**Figure 16:**
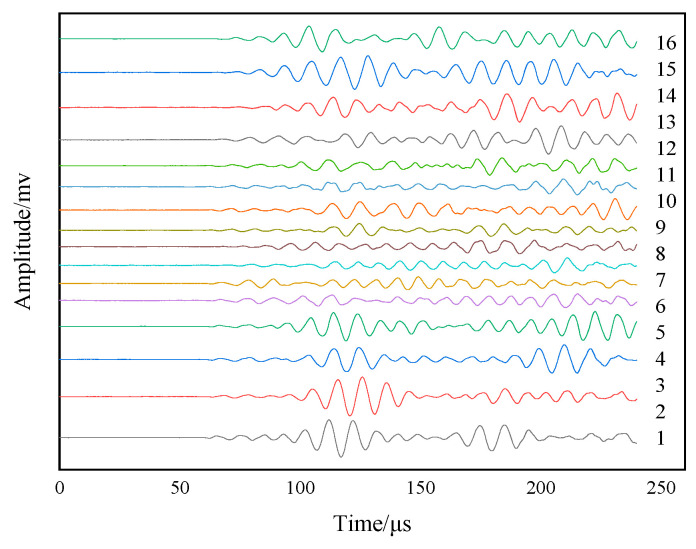
Reconstructed signal in the experiment.

Firstly, the initial Lamb wave excitation signal is emitted by the excitation sensor at the center of the plate, and the response signal is received by PZT1–PZT16. Then, the response signal of PZT1 is selected, and the wave band within 0.000127 s is extracted for time domain inversion to obtain the time-reversal excitation signal. Finally, the time-reversal excitation signal is emitted by the excitation sensor, and PZT1 to PZT16 receive the signal again to obtain the Lamb wave reconstruction signal, as shown in Figure 16.

**Figure 17:**
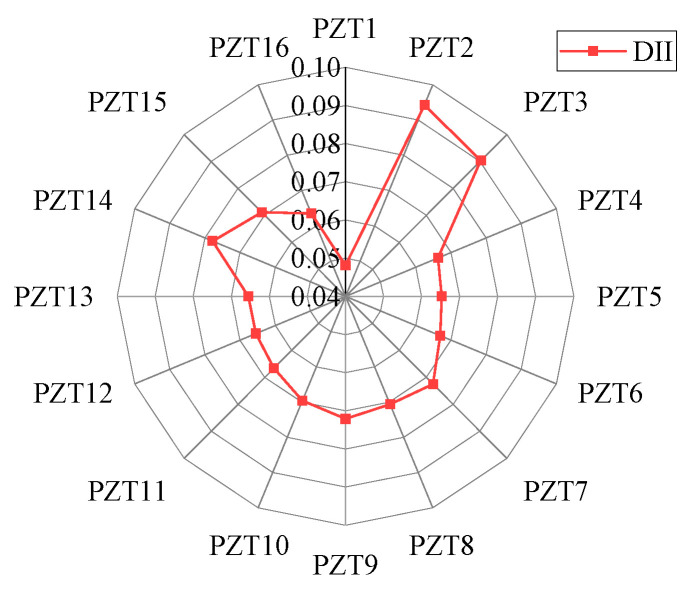
Difference indices of each sensing path in the experiment.

**Figure 18:**
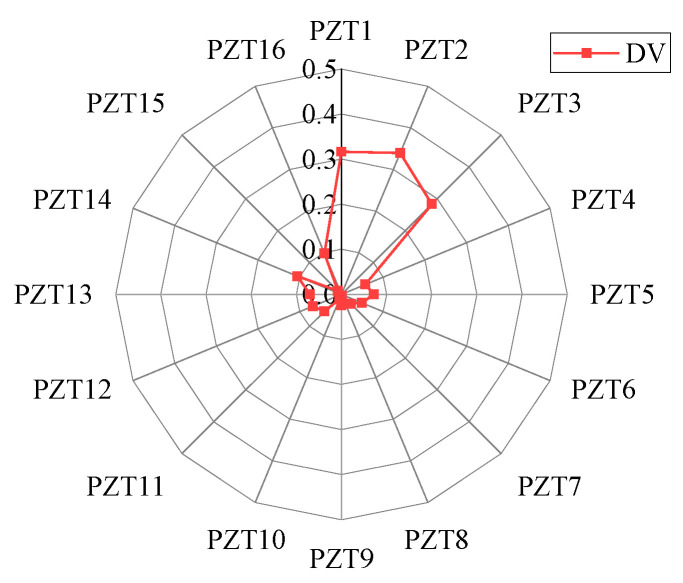
The deviation values of each sensing path in the experiment.

The focused main wave packet is analyzed, and the difference indices of sensing path 1 to path 16 are calculated according to Formula (7), which reflects the difference between the focused main wave packet and the initial excitation signal on each sensing path, as shown in Figure 17. In Figure 17, the difference index of the sensing paths is about 0.07 except for sensing paths 1, 2, and 3, so the focused main wave packet of sensing paths 1, 2, and 3 is quite different from that of other sensing paths. The deviation value of sensing path 1 to path 16 is calculated by Formula (8), and the deviation degree of the signal on the sensing path is quantified more accurately, as shown in Figure 18.

**Figure 19:**
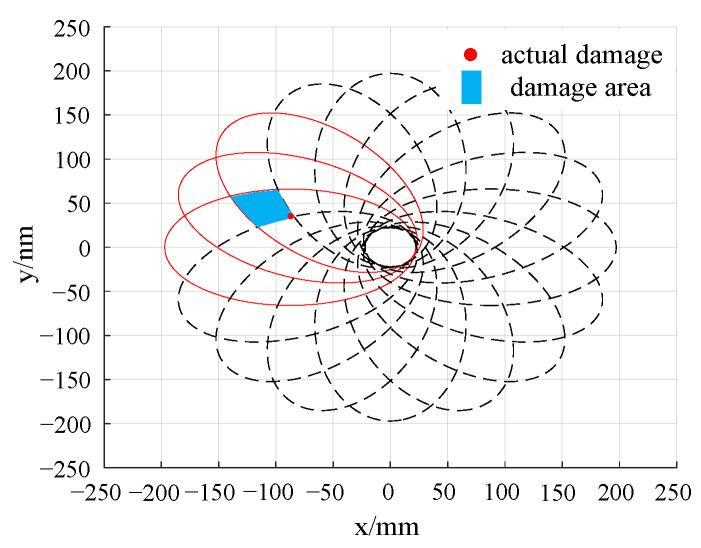
Damage location results using deviation values in the experiment.

According to the deviation values of each sensing path shown in Figure 18, it can be clearly observed that the deviation values of sensing paths 1, 2, and 3 are significantly larger than those of other sensing paths. In the process of damage identification, it is generally believed that the sensing path with a larger deviation is more likely to be close to the location of damage. Therefore, it can be preliminarily inferred that the damage is located in the intersection of the damage identification range of paths 1, 2, and 3 and is not within the damage identification range of other sensing paths, as shown in Figure 19.

### 4.2. Probabilistic Damage Imaging in the Experiment

**Figure 20:**
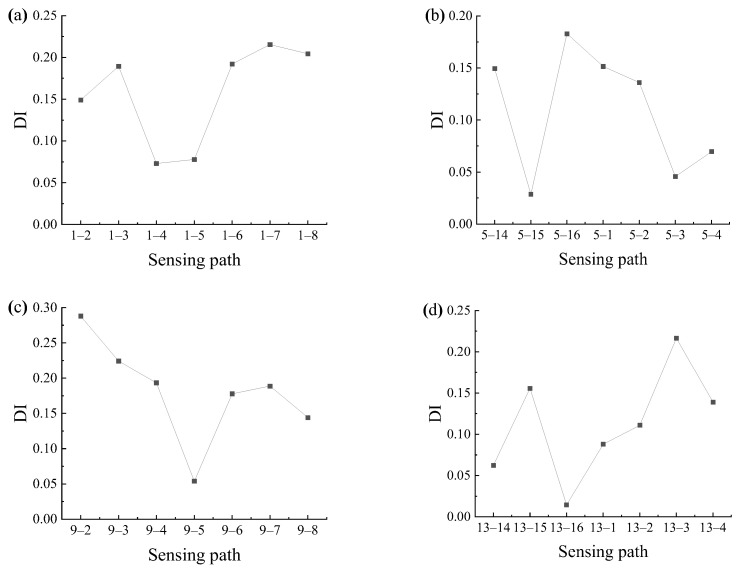
Damage indices of each sensing path emitted by different sensors in the experiment: (**a**) PZT1; (**b**) PZT5; (**c**) PZT9; (**d**) PZT13.

To further locate the damage in the measured structure, Lamb wave signals of A0 mode are emitted at four different sensors, PZT1, PZT5, PZT9, and PZT13, respectively. These four sensors are distributed in different regions of the plate, which helps to obtain more comprehensive and accurate damage information. After the Lamb wave signals are emitted by PZT1, PZT5, PZT9, and PZT13, respectively, other receiving sensors are used to receive and record the Lamb wave signals. To reduce the influence of noise in the experiment and better extract the damage information, the signal of length (5) is selected to analyze. The intercepted signal is analyzed by using Formula (9), and the damage index of each sensing path is calculated, as shown in Figure 20.

**Figure 21:**
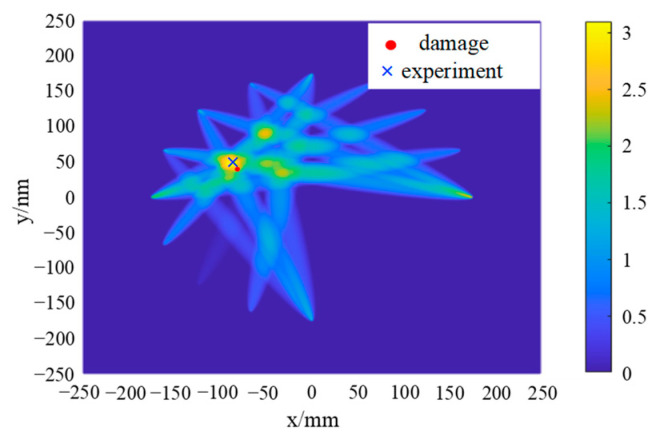
Probabilistic damage imaging results in the experiment.

Figure 20 provides a clear visualization of the distribution of damage indices along various sensing paths. Notably, the sensing paths with the largest damage indices are still paths 1–7, 5–16, 9–2, and 13–3, which is consistent with the numerical simulation results, indicating that the damage may be located near these sensing paths. However, due to the inevitable influence of noise and structural inhomogeneity during the experiment, the damage index of sensing paths shows a relatively large value in the experiment. The damage indices of sensing paths passing through the second quadrant are selected for the damage probability imaging, and the damage imaging results are shown in Figure 21.

As can be seen from Figure 21, the highlighted area in the damage imaging results represents the region with the highest probability of damage, and its center position is (−89.50, 39.50). Compared with the actual damage position (−87.29, 36.16), the error in the *x* direction is 2.53%, and the error in the *y* direction is 9.24%. When comparing the experimental damage location results with the numerical simulation results, while the position with the highest damage probability still corresponds to the actual damage location, other regions with relatively high damage probability also emerge. The maximum damage probability path in the simulation is path 13–3, while the path with the largest damage probability in the experiment becomes path 9–2. This shift can be attributed to the combined effects of various factors in the experiment, such as noise, material inhomogeneity, structural complexity, and more. These factors can influence the propagation and reception of Lamb waves, ultimately affecting the accuracy of the final damage location.

## 5. Discussion

Through theoretical analysis of the PEX-TRM, it can be discovered that the migration degree of the damage wave packet in the reconstructed signal has a direct correlation with the position of the sensor receiving the reconstructed signal. When the damage location is close to the sensing path, the aliasing phenomenon between the damage wave packet and the focused main wave packet becomes particularly pronounced, directly impacting the waveform characteristics of the focused main wave packet.

In the process of using different receiving sensors to obtain the response signal and then generate the time-reversal excitation signal, although the data from different position sensors are used, the difference index of each sensing path of the reconstructed signal is the same. This similarity is consistent with the theoretical derivation that the degree of aliasing between the main wave packet and the damage wave packet in the reconstructed signal depends only on the location of the damage and the position of the receiving sensor rather than the specific source of the time-reversal excitation signal. Therefore, in the PEX-TRM, the response signal of any receiving sensor can be flexibly selected to generate the time-reversal excitation signal.

When analyzing the change of the focused main wave packet, it can be found that when a sensing path is located near the damage, the deviation value will be significantly higher than other paths. According to the number and location of the sensing paths affected by the damage, the structure can be divided into multiple damage units to determine the location of the damage. And as the density of the sensing path increases, the divided damage units will be more detailed, and the accuracy of damage identification will also be improved.

When using probabilistic damage imaging technology to further locate damage, the selection and interception of signals become crucial. To ensure the accuracy of damage identification, it is essential to intercept the appropriate signal segment to calculate the damage index. The damage information contained in other wave packets after the direct wave will reduce the accuracy of the method. In the experiment, the zero-amplitude band signal segment before the direct wave will also produce interference due to the influence of noise, so only the direct wave signal should be intercepted as far as possible for analysis.

## 6. Conclusions

This paper proposes a Lamb wave probabilistic damage identification method based on the focused main wave packet of the reconstructed signal in the EX-TRM. This method no longer needs to extract the damage wave packet, which reduces the requirement for signal quality, facilitates damage identification, and is more suitable for practical engineering. Through theoretical analyses, simulation analyses, and experiments, the following conclusions are drawn:

1. The theoretical analysis reveals that the closer the sensing path is to the damage, the closer it is between the damage wave packet and the focused main wave packet in the reconstructed signal, the more obvious the waveform change of the focused main wave packet, and the larger the deviation value, and it is independent of the specific source of the time-reversal excitation signal.
2. By calculating the relative deviation value of each sensing path to analyze the number and location of the sensing paths affected by the damage, the structural damage can be divided into the corresponding regional units of the damage, thereby realizing the damage location. And the denser the sensing paths, the smaller the divided region unit, and the more accurate the damage location.
3. For regions with sparse sensing paths, damage probability imaging can be used to further locate the damage. Based on a rough determination of the damage location, only sensing paths passing through the known damage area are used for damage imaging, and the correlation of the symmetrical sensing paths is calculated. This approach not only accurately locates the damage but also effectively reduces the number of required excitations, avoiding a large amount of invalid sensing path data and improving the efficiency of damage identification.
4. The PEX-TRM can obtain a large amount of damage data with fewer excitations for damage location, and it further improves the efficiency of damage identification because it no longer needs to extract the damage wave packet, which is more conducive to engineering application. Future research will be conducted on its application in complex structures and working conditions, with a focus on the severity and classification of damage, aiming to achieve a higher level of damage identification hierarchy and further promote the practical application of this method.

## Figures and Tables

**Table 1:** The coordinates of receiving sensors (mm).

Sensor	PZT1	PZT2	PZT3	PZT4
Coordinates	(−175.00, 0.00)	(−161.68, 66.97)	(−123.74, 123.74)	(−66.97, 161.68)
Sensor	PZT5	PZT6	PZT7	PZT8
Coordinates	(0.00, 175.00)	(66.97, 161.68)	(123.74, 123.74)	(161.68, 66.97)
Sensor	PZT9	PZT10	PZT11	PZT12
Coordinates	(175.00, 0.00)	(161.68, −66.97)	(123.74, −123.74)	(66.97, −161.68)
Sensor	PZT13	PZT14	PZT15	PZT16
Coordinates	(0.00, −175.00)	(−66.97, −161.68)	(−123.74, −123.74)	(−161.68, −66.97)

**Table 2:** The coordinates of damages (mm).

Damage	1	2	3	4
Coordinates	(−29.42, 5.85)	(−58.85, 11.71)	(−88.27, 17.56)	(−117.69, 23.41)
Damage	5	6	7	8
Coordinates	(−120.00, 0.00)	(−119.42, 11.76)	(−114.83, 34.83)	(−110.87, 45.92)
Damage	9	10	11	12
Coordinates	(−105.83, 56.57)	(−99.78, 66.67)	(−92.76, 76.13)	(−84.85, 84.85)

## Data Availability

The data presented in this study are available on request from the corresponding author. The data are not publicly available due to ethics restrictions on data dissemination and storage.

## References

[1] Chen X., Lu Y.G., Li Z.W., Cui Z.H. (2024). Experimental investigation of spectral evolution in flash radiation by hypervelocity impact on aluminum plates. Def. Technol..

[2] Song Y.C., Zhang M.Y., Ke K., Yam M.C.H., Lin X.M. (2023). Behaviour and design of gusset plates in steel structures: A state-of-the-art review. J. Constr. Steel Res..

[3] Ling T.T., Mohrmann S., Li H.T., Bao N.Z., Gaff M., Lorenzo R. (2022). Review on research progress of metal-plate-connected wood joints. J. Build. Eng..

[4] Riahi H., Bressolette P., Chateauneuf A. (2010). Random fatigue crack growth in mixed mode by stochastic collocation method. Eng. Fract. Mech..

[5] Zhu S.P., Ai Y., Liao D., Correia J.A.F.O., De Jesus A.M.P., Wang Q.Y. (2021). Recent advances on size effect in metal fatigue under defects: A review. Int. J. Fract..

[6] Radgolchin M., Anbarsooz M. (2023). Fatigue failure of centrifugal compressor impellers: A comprehensive review. Eng. Fail. Anal..

[7] Ostachowicz W., Soman R., Malinowski P. (2019). Optimization of sensor placement for structural health monitoring: A review. Struct. Health Monit..

[8] Bratov V.A., Kuznetsov S.V., Morozov N.F. (2022). Lamb problems and related problems in dynamics: A review. Mech. Solids.

[9] Hu M.P., He J., Zhou C., Shu Z.Y., Yang W.P. (2021). Surface damage detection of steel plate with different depths based on Lamb wave. Measurement.

[10] Zheng S.P., Luo Y., Xu C.G., Xu G.D. (2023). A review of laser ultrasonic Lamb wave damage detection methods for thin-walled structures. Sensors.

[11] Ono K. (2022). Experimental determination of Lamb-wave attenuation coefficients. Appl. Sci..

[12] Yu Q.P., Zhou S.Y., Cheng Y.H., Deng Y. (2024). Research on delamination damage quantification detection of CFRP bending plate based on Lamb wave mode control. Sensors.

[13] Gorgin R., Luo Y., Wu Z.J. (2020). Environmental and operational conditions effects on Lamb wave based structural health monitoring systems: A review. Ultrasonics.

[14] Lu H.Y., Chandran B., Wu W., Ninic J., Gryllias K., Chronopoulos D. (2024). Damage features for structural health monitoring based on ultrasonic Lamb waves: Evaluation criteria, survey of recent work and outlook. Measurement.

[15] Altammar H., Khan M.F. (2024). Evaluation of welded lap joints using ultrasonic guided waves. Sensors.

[16] Yun H.G., Rayhana R., Pant S., Genest M., Liu Z. (2021). Nonlinear ultrasonic testing and data analytics for damage characterization: A review. Measurement.

[17] Su C.H., Jiang M.S., Liang J.Y., Tian A.Q., Sun L., Zhang L., Zhang F.Y., Sui Q.M. (2020). Damage location of composites based on difference signal and Lamb wave tomography. Materials.

[18] Andrews J.P., Palazotto A.N., DeSimio M.P., Olson S.E. (2008). Lamb wave propagation in varying isothermal environments. Struct. Health Monit.—Int. J..

[19] Xu C.B., Wang J.S., Yin S.X., Deng M.X. (2021). A focusing MUSIC algorithm for baseline-free Lamb wave damage location. Mech. Syst. Signal Process..

[20] Sun H., Yi J., Xu Y., Wang Y., Qing X. (2019). Identification and Compensation Technique of Non-Uniform Temperature Field for Lamb Wave-and Multiple Sensors-Based Damage Detection. Sensors.

[21] Wee J., Alexander K., Peters K. (2021). Self-referencing ultrasound detection of fiber Bragg grating sensor with two adhesive bonds. Meas. Sci. Technol..

[22] Marasco G., Oldani F., Chiaia B., Ventura G., Dominici F., Rossi C., Iacobini F., Vecchi A. (2022). Machine learning approach to the safety assessment of a prestressed concrete railway bridge. Struct. Infrastruct. Eng..

[23] Ambrozinski L., Stepinski T., Packo P., Uhl T. (2012). Self-focusing Lamb waves based on the decomposition of the time-reversal operator using time–frequency representation. Mech. Syst. Signal Process..

[24] Yu S.Q., Fan C.G., Zhang M., Zhao Y. (2023). A Lamb wave time-reversal field reconstruction method for damage detection with automatic focusing determination. Ultrasonics.

[25] Jeong H.J., Cho S.J., Wei W. (2011). A baseline-free defect imaging technique in plates using time reversal of Lamb waves. Chin. Phys. Lett..

[26] Wang B.Q., Shi W.J., Zhao B., Tan J.B. (2022). A modal decomposition imaging algorithm for ultrasonic detection of delamination defects in carbon fiber composite plates using air-coupled Lamb waves. Measurement.

[27] Bijudas C.R., Mitra M., Mujumdar P.M. (2013). Time reversed Lamb wave for damage detection in a stiffened aluminum plate. Smart Mater. Struct..

[28] Huo H.D., He J.J., Guan X.F. (2020). A Bayesian fusion method for composite damage identification using Lamb wave. Struct. Health Monit.—Int. J..

[29] Zhao X., Royer R.L., Owens S.E. (2011). Ultrasonic Lamb wave tomography in structural health monitoring. Smart Mater. Struct..

[30] Xia Q.W., Liu Y.Y., Lu Y., Cao S.H., Zhang H.F., Ma S.W. (2019). A modified damage index probability imaging algorithm based on delay-and-sum imaging for synthesizing time-reversed Lamb waves. J. Vibroeng..

[31] Miao X.T., Wang D., Ye L., Lu Y., Li F.C., Meng G. (2011). Identification of dual notches based on time-reversal Lamb waves and a damage diagnostic imaging algorithm. J. Intell. Mater. Syst. Struct..

[32] Liu Z.H., Zhong X.W., Dong T.C., He C.F., Wu B. (2017). Delamination detection in composite plates by synthesizing time-reversed Lamb waves and a modified damage imaging algorithm based on RAPID. Struct. Control. Health Monit..

[33] Park H.W., Kim S.B., Sohn H. (2009). Understanding a time reversal process in Lamb wave propagation. Wave Motion.

[34] Duan Q.M., Ye B., Zou Y.K., Hua R., Feng J.Q., Shi X.X. (2023). Probability-based diagnostic imaging of fatigue damage in carbon fiber composites using sparse representation of Lamb waves. Electronics.

[35] Cai J., Shi L.H., Yuan S.F., Shao Z.X. (2011). High spatial resolution imaging for structural health monitoring based on virtual time reversal. Smart Mater. Struct..

[36] Watkins R., Jha R. (2012). A modified time reversal method for Lamb wave based diagnostics of composite structures. Mech. Syst. Signal Process..

[37] Gao Y.M., Sun L.Y., Song R.J., Peng C., Wu X.B., Wei J.T., Jiang M.S., Sui Q.M., Zhang L. (2024). Damage location in composite structures based on Lamb wave and modular artificial neural network. Sens. Actuators A Phys..

[38] Shu Z.Y., He J., Hu M.P., Zhou C., Sun X.D. (2023). Damage Location of Plate Structures based on the Exchanging-element Time-reversal Method by using Lamb Waves. J. Sound Vib..

[39] Wang D., Ye L., Lu Y. (2009). Probabilistic Diagnostic Algorithm for Identification of Multiple Notches Using Digital Damage Fingerprints (DDFs). J. Intell. Mater. Syst. Struct..

[40] Ling F.Y., Chen H.L., Lang Y.F., Yang Z.B., Xu K.L., Ta D.A. (2023). Lamb wave tomography for defect location using wideband dispersion reversal method. Measurement.

